# Economic evaluations of digital health interventions on maternal, newborn and child health in low-income and middle-income countries: a systematic review protocol

**DOI:** 10.1136/bmjopen-2025-115990

**Published:** 2026-05-07

**Authors:** Fei Wu, Brenda Soun, Binqi Zhan, Alec Morton, Siyan Yi

**Affiliations:** 1 National University Health System, Saw Swee Hock School of Public Health, National University of Singapore, Singapore; 2 United World College of South East Asia - Dover Campus, Singapore; 3 Duke-NUS Medical School, Singapore; 4 University of Strathclyde, Glasgow, UK; 5 KHANA Center for Population Health Research, Phnom Penh, Cambodia; 6 Public Health Program, College of Education and Health Sciences, Touro University California, Vallejo, California, USA

**Keywords:** Digital Technology, Mothers, Child

## Abstract

**Abstract:**

**Introduction:**

Digital health interventions have emerged as promising solutions to strengthen maternal, newborn and child health (MNCH) systems in low-income and middle-income countries (LMICs). Although evidence on their effectiveness is growing, corresponding economic evaluations remain limited, heterogeneous and fragmented. Understanding the value for money of digital health interventions is essential for supporting scale-up decisions and efficient resource allocation. This protocol outlines the methods for a systematic review synthesising economic evaluations of digital health interventions across the MNCH continuum in LMICs.

**Methods and analysis:**

We will systematically search PubMed, Embase, Scopus, the Cochrane Library, Web of Science, Cumulative Index to Nursing and Allied Health Literature (CINAHL), PsycINFO and Turning Research Into Practice (TRIP) Pro for peer-reviewed studies published from 1 January 2000 to 31 December 2025. Backward citation searching (reference lists of included articles) and forward citation tracking will be performed to ensure comprehensive study identification, and study authors will be contacted when necessary to obtain missing or unclear data. Artificial intelligence (AI)-based Deep Research tools will be used as a supplementary approach to identify additional keywords and assess the completeness of the search strategy. Eligible studies will include full and partial economic evaluations embedded within randomised controlled trials, quasi-experiments, controlled before–and–after studies, time-series analyses, cohort or case–control designs, implementation studies and economic modelling studies. Three reviewers will independently conduct study selection and data extraction using a predefined form adapted from the Consolidated Health Economic Evaluation Reporting Standards (CHEERS) 2022 guidelines. Methodological quality will be assessed using the Drummond checklist. Due to heterogeneity in study designs and economic methods, a narrative synthesis will be conducted. Costs will be standardised to the US dollar (USD) in 2024.

**Ethics and dissemination:**

This review will synthesise evidence from already published studies and does not involve the collection of primary data; therefore, ethical approval is not required. All data extracted will be derived from publicly available peer-reviewed literature, and no identifiable personal information will be used. The findings of this systematic review will be disseminated through publication in a peer-reviewed journal and presentations at academic conferences focused on global health, digital health and MNCH. In addition, the results will be shared with relevant stakeholders, including policymakers, programme implementers and organisations involved in digital health and MNCH in LMICs.

**PROSPERO registration number:**

CRD420251125682.

**STRENGTHS AND LIMITATIONS OF THIS STUDY**

- A comprehensive search strategy will be employed across eight electronic databases covering the period from 2000 to 2025, with database-specific adaptations to capture variations in indexing systems and terminology, to maximise the sensitivity and completeness of study identification.
- Incorporation of an artificial intelligence(AI)-assisted approach to refine search terminology and assess search completeness, addressing challenges related to inconsistent digital health terminology.
- Inclusion of both full and partial economic evaluations across a wide range of study designs, allowing broad coverage of the evidence of the cost-effectiveness of digital health interventions on maternal, newborn and child health in low-income and middle-income countries.
- Heterogeneity in intervention types, economic methods and contexts may limit comparability and preclude pooling of results or meta-analyses.
- Reliance on published peer-reviewed literature alone could lead to publication bias and the exclusion of unpublished or grey literature on economic evaluations, potentially under-representing less favourable or null results.

## Background

 Maternal, newborn and child health (MNCH) remains a global priority, particularly in low-income and middle-income countries (LMICs), where preventable morbidity and mortality remain unacceptably high. In 2023, approximately 260 000 women died from pregnancy-related causes, with 92% occurring in LMICs.^1^ Neonatal mortality accounted for 2.3 million deaths, representing nearly half of under-five mortality.^2^ Despite global progress in MNCH over recent decades, many LMICs continue to face significant system-level barriers, including limited health workforce capacity, geographic inequities in access to care, supply chain gaps and a fragmented health information system.^3^ These systemic failures disproportionately affect vulnerable populations, demanding innovative, scalable and efficient solutions.

Digital health interventions, including mobile health (mHealth), electronic health (eHealth), telehealth and digital decision-support tools, have emerged as promising strategies to strengthen MNCH service delivery.^4 5^ The WHO Global Strategy on Digital Health 2020–2025 highlights the potential of digital technologies to enhance service coverage, support frontline health workers, optimise supply chains and empower beneficiaries with health information.^6^ Digital health solutions span a spectrum of functions: short message service (SMS) and interactive voice response (IVR) messaging for client engagement, mobile applications for community health workers, telemedicine for maternal and neonatal emergencies, digital logistics systems for immunisation, and electronic registries for pregnancy surveillance and newborn tracking.^7^,^9^

There is increasing evidence that digital health interventions can improve MNCH outcomes. For example, mobile messaging has been linked to improved antenatal care attendance, breastfeeding practices and postpartum contraception.^10^,^12^ Digital job aids and decision-support applications enhance the quality of care delivered by frontline workers.^13 14^ Telemedicine models have demonstrated increased access to neonatal care and specialist support.^15 16^ Moreover, supply chain innovations, such as real-time vaccine logistics systems, have been shown to reduce stockouts and improve immunisation coverage.^17^ However, despite these promises, digital health interventions vary widely in implementation scale, from small pilot projects to national platforms, leading to variation in costs, cost-effectiveness and scalability.^18 19^ The successful implementation of a digital intervention is highly dependent on contextual factors, including existing infrastructure, health worker literacy, digital access and local government buy-in, all of which introduce complexity and variability into the actual economic burden and realised benefits.^20^

Economic evaluation methods, such as cost-effectiveness analysis (CEA), are essential tools in health-sector priority setting, grounded in principles of constrained optimisation to maximise population health gains within limited resources.^21^ Although evidence on the effectiveness of digital health interventions for MNCH has grown substantially in recent years, the corresponding economic evidence remains limited, fragmented and methodologically heterogeneous.^18^ Few studies have systematically examined economic evaluations of digital health interventions, either specific to MNCH or focused on LMICs. Yet such assessments are essential for guiding investment decisions, informing scale-up strategies and ensuring the efficient allocation of constrained health budgets, despite the need for cost-effective solutions to strengthen health systems. Also, inconsistencies in digital health terminology and substantial variation in intervention design complicate the systematic identification and synthesis of relevant economic evidence.

Given these gaps, a systematic review of economic evaluations of digital health interventions across the MNCH continuum in LMICs is warranted. This systematic review will synthesise the financial assessments of digital health interventions for MNCH in LMICs. In particular, it aims to determine the cost-effectiveness of digital health interventions compared with standard care or non-digital alternatives, to identify which types of digital health strategies offer the most significant economic value across diverse MNCH conditions and implementation contexts, and to examine the key factors that influence costs and cost-effectiveness, including intervention design, delivery platform, implementation scale and broader health system characteristics. By consolidating existing evidence, the review seeks to identify methodological and contextual gaps, compare the value of different digital health approaches and generate actionable insights to help policymakers and donors prioritise cost-effective, scalable digital solutions that can strengthen MNCH outcomes in resource-constrained settings.

## Methods

This systematic review follows the Preferred Reporting Items for Systematic Reviews and Meta-Analyses (PRISMA) statement,^22^ and the Preferred Reporting Items for Systematic Review and Meta-Analysis Protocols PRISMA-P checklist is provided (online supplemental material A). A detailed protocol has been prospectively registered with the International Prospective Register for Systematic Reviews (PROSPERO ID: CRD420251125682).

### Data source and search strategy

We will search for published, peer-reviewed literature in the following eight electronic databases: PubMed, Embase (Ovid), Scopus, the Cochrane Library, Web of Science, Cumulative Index to Nursing and Allied Health Literature (CINAHL), PsycINFO and Turning Research Into Practice (TRIP) Pro version. Searches will cover articles published from January 2000 to the end of 2025. Further studies will be identified through backward citation searching of reference lists and forward citation tracking of included articles. When necessary, study authors will be contacted to obtain missing information or clarify unclear data.

The search strategy will be developed using a combination of controlled vocabulary and keywords for digital health and MNCH concepts. The initial strategy will be developed in PubMed and subsequently adapted for each database to account for differences in indexing systems, controlled vocabularies and search interfaces. Database-specific subject headings will be used where appropriate, including Medical Subject Headings (MeSH) in PubMed, Emtree terms in Embase and CINAHL Headings in CINAHL. In addition, database-specific field tags, Boolean operators, truncation symbols and proximity operators will be applied in accordance with each platform’s requirements. The complete search strategies for all databases are provided in online supplemental material B to ensure transparency and reproducibility.

To support the development and refinement of the search strategy, a generative AI tool (ChatGPT, OpenAI) will be used to identify additional keywords, synonyms and related concepts relevant to the review topic. The AI tool will be used as a supplementary aid during the search strategy development phase and will not replace structured searches of bibliographic databases. Predefined prompts will be used to generate potential search terms, and all AI-generated suggestions will be reviewed and validated by the research team before being incorporated into the final search strategy. As an additional verification step, the AI tool will be used to conduct a parallel exploratory search to identify potentially relevant studies and assess whether the database search strategy would have missed any eligible articles; any suggested studies will be subsequently verified and retrieved through the structured database searches. The AI tool will not be used for study screening or selection. To ensure transparency and reproducibility, representative prompts and the workflow used for AI-assisted search development are provided in online supplemental material C.

### Inclusion and exclusion criteria

#### Study design

This review will include both full and partial economic evaluations embedded within eligible study designs. Acceptable designs will comprise randomised controlled trials and their variants, quasi-experimental studies, controlled before-and-after studies, interrupted time-series analyses, cohort and case-control studies with economic evaluation components, and decision-analytic or modelling studies such as Markov or simulation models. Implementation studies that include cost or economic components will also be eligible.

Descriptive studies without economic data, systematic reviews, meta-analyses, narrative reviews, commentaries and editorials will be excluded. Conference abstracts and grey literature will be excluded because they often provide limited methodological information and incomplete economic outcome data, which may affect the reliability and comparability of the evidence.

#### Population

Eligible studies must involve populations relevant to MNCH, including reproductive-age women, pregnant women, postpartum women up to 12 months after delivery, newborns aged 0–28 days, infants up to 12 months and children under 5 years of age.

#### Intervention

The review will include studies that evaluate digital health interventions designed to improve MNCH outcomes, including but not limited to: (1) mobile phone-based interventions (SMS/text messaging, voice calls, interactive voice response), (2) smartphone/tablet applications (apps for education, remote monitoring or decision support), (3) wearable devices (eg, for maternal or infant health tracking), (4) telemedicine/teleconsultation platforms for MNCH care and (5) electronic health records. Interventions without a clear MNCH focus will be excluded.

#### Comparator

Comparators of interest will include standard care, non-digital or paper-based alternatives, historical or pre-intervention conditions in before-and-after studies, or alternative digital strategies when applicable. Studies without a comparator will be included if they provide economic evaluations such as cost analyses.

#### Outcomes

The primary outcomes of interest for this systematic review will include economic outcomes that quantify the value-for-money of digital health interventions, including incremental cost-effectiveness ratios (ICERs), such as cost per disability-adjusted life year (DALY) averted, cost per life saved or cost per additional service or health outcome achieved; cost-benefit ratios; net monetary benefit and other summary measures of economic value. Where available, we will extract the willingness-to-pay thresholds applied in individual studies to aid the interpretation of cost-effectiveness findings. Secondary outcomes will include total and incremental programme costs, cost per beneficiary reached, cost per contact or service delivered and contextual cost drivers such as personnel, technology and implementation costs.

#### Context

Only studies conducted in LMICs, as classified by the World Bank at the time when the primary studies were undertaken, will be included. No restrictions will be placed on the delivery setting; studies conducted in community-based, facility-based or hybrid models will all be eligible. Only studies published in English will be included.

### Study selection

All retrieved citations will be imported into Covidence (Veritas Health Innovation Ltd., Melbourne, Australia) for automatic duplicate removal and subsequent screening. Study selection will occur in two stages. First, titles and abstracts will be screened independently by two reviewers from the three-member review team (FW, BZ and BS) to exclude clearly ineligible studies. Second, full-text articles for all potentially eligible records will be retrieved and assessed independently by two reviewers against the predefined inclusion and exclusion criteria. Any disagreements at either stage will be resolved through discussion and, if necessary, consultation with a senior reviewer. Reasons for exclusion at the full-text stage will be documented within Covidence and reported in the PRISMA flow diagram.

### Data extraction

Data extraction will be conducted using a standardised Excel form, adapted from the Consolidated Health Economic Evaluation Reporting Standards (CHEERS) 2022 reporting guidelines.^23^ The form will be piloted on a subset of included studies and refined as needed. Two reviewers will independently extract data from all included studies. Extracted variables will consist of citation details, study setting (country and World Bank income level), study design, economic analytic approach, time horizon, currency, costing year, discount rate, population characteristics, intervention details (including digital functions and delivery technologies), comparator, cost outcomes and cost-effectiveness outcomes. The extraction files will be compared for consistency, and discrepancies will be resolved through discussion and re-examination of the full text. For studies with incomplete or unclear information on key variables (eg, cost data, outcome measures or methodological details), one attempt will be made to contact the corresponding author via email. All final extracted data will be stored securely and archived for transparency and reproducibility.

### Quality and bias assessment

Methodological quality of the included economic evaluations will be assessed using the Drummond Checklist, which evaluates 10 key domains of economic evaluation quality, including clarity of the research questions, description of alternatives, establishment of effectiveness, identification and measurement of costs and consequences, evaluation methods, discounting, incremental analyses, handling of uncertainty and generalisability.^24^ Each item will be scored using the Doran adaptation, assigning a value of 1 when a criterion is adequately met and 0 otherwise, yielding a total score of 0 to 10. Studies will be classified as good (8–10 points), average (4–7 points) or poor (1–3 points) in methodological quality.^25^

All assessments will be conducted independently by two reviewers, with discrepancies resolved through discussion or consultation with a senior reviewer. When required information is unclear or missing from published reports, we will contact study authors for clarification.

### Strategy for data synthesis

Given the anticipated heterogeneity in study designs, types of digital health interventions and economic evaluation methods, a formal meta-analysis is not feasible. Furthermore, a quantitative synthesis of the primary economic outcome, ICER, is statistically inappropriate. Instead, we will undertake a structured narrative synthesis of the included economic evaluations. This synthesis will follow established guidance on the qualitative integration of cost and cost-effectiveness evidence in systematic reviews, including grouping studies by intervention function, MNCH domain, implementation scale, type of economic evaluation and analytic methods. Where feasible, interventions will be categorised into thematic groups, including communication-based tools (SMS, IVR), decision-support applications, telemedicine platforms, digital data systems and supply-chain/logistics technologies.

To facilitate comparability across studies conducted in different countries, cost data will be standardised to a common currency (US dollars, USD) and a common price year. Costs reported in different years will first be adjusted for inflation using country-specific inflation indices, such as the Consumer Price Index obtained from the World Bank. The year 2024 was selected as it is the most recent year with available inflation data from the World Bank. The adjusted cost values will then be converted to USD using exchange rates from the International Financial Statistics database.

The primary outcomes of interest will include cost measures (eg, total and per-participant costs) and cost-effectiveness outcomes, particularly ICERs expressed in terms such as cost per DALY averted, quality-adjusted life year (QALY) gained, or life-year saved. Given the expected heterogeneity in economic evaluation methods, outcome measures and study contexts, cost-effectiveness results will be synthesised narratively rather than quantitatively. Where feasible, ICERs will be compared within studies reporting similar outcome measures (eg, cost per DALY averted or cost per QALY gained), and interpreted in relation to commonly used cost-effectiveness thresholds.

To support comparison across studies and interpret the direction and magnitude of economic results, we will classify interventions using a permutation matrix adapted from the Joanna Briggs Institute Guidelines for Systematic Reviews of Economic Evaluations.^26^ Specifically, each intervention will be assessed according to whether it demonstrates higher, lower or similar costs and effects relative to the comparator. Findings from the narrative synthesis will be complemented by tables summarising study characteristics, cost outcomes, cost-effectiveness outcomes and quality assessments.

## Discussion

With rapid advances in digital technologies, digital health interventions have become increasingly prominent as innovative solutions to strengthen MNCH systems, particularly in LMICs where persistent resource constraints limit coverage, quality and continuity of care.^4 5^ While the effectiveness of such interventions has been documented across a range of MNCH outcomes, including improved service utilisation, enhanced quality of care and greater access to specialist support,^18^ the corresponding economic evidence remains comparatively sparse, fragmented and heterogeneous. This systematic review will address this critical gap by comprehensively synthesising full and partial economic evaluations of digital health interventions across the MNCH continuum in LMICs.

By examining reported cost outcomes, the review will support future programme planners in developing more accurate budget projections and identifying key cost drivers that influence financial sustainability. By synthesising cost-effectiveness metrics and contextual determinants of economic performance, the review will generate insights into which digital health strategies offer the most significant value for money and under what implementation conditions. Identifying persistent methodological limitations, such as limited uncertainty analyses, inconsistent cost reporting and variability in analytic perspectives, will help guide priorities for strengthening the rigour and comparability of future economic evaluations of digital health interventions.

The findings of this review will have direct implications for policy and programme decision-making. As LMIC health systems continue to expand investments in digital technologies, evidence regarding their economic value is essential to ensure sustainable financing, equitable implementation and effective scale-up. By identifying intervention types and implementation models with favourable cost-effectiveness profiles, this review will support policymakers, donors and programme implementers in making informed resource allocation decisions and prioritising digital interventions that deliver the most significant and most cost-effective improvements in MNCH outcomes.

### Limitations

This review may face several methodological and contextual limitations. First, substantial heterogeneity is expected across the included studies in intervention types, costing approaches, analytic perspectives, outcome measures and implementation contexts. Such variability will limit comparability and preclude quantitative meta-analysis, requiring reliance on narrative synthesis. Second, economic evaluations are often influenced by contextual factors such as health system structures, population characteristics and local resource prices. This may reduce the transferability of findings across LMIC settings, and contextual differences may not be fully captured in published reports. Third, because we will include only peer-reviewed literature and exclude grey literature and conference abstracts, there is a risk of publication bias, particularly if evaluations reporting unfavourable or inconclusive cost-effectiveness results are less likely to be published.

## Supplementary material

10.1136/bmjopen-2025-115990 online supplemental file 1

10.1136/bmjopen-2025-115990 online supplemental file 2

10.1136/bmjopen-2025-115990 online supplemental file 3

## References

[1] (2025). Trends in maternal mortality estimates 2000 to 2023: estimates by WHO, UNICEF, UNFPA, World Bank Group and UNDESA/Population Division.

[2] United Nations Inter-agency Group for Child Mortality Estimation (2025). Levels & Trends in Child Mortality 2024. United Nations Children’s Fund. https://data.unicef.org/resources/levels-and-trends-in-child-mortality-2024.

[3] Duwiejua M, Steele P, Lalvani P (2025). Promising Practices in Capacity Development for Health Supply Chains in Resource-Constrained Countries. Glob Health Sci Pract.

[4] Agarwal S, Perry HB, Long L (2015). Evidence on feasibility and effective use of mH ealth strategies by frontline health workers in developing countries: systematic review. Tropical Med Int Health.

[5] Labrique AB, Vasudevan L, Kochi E (2013). mHealth innovations as health system strengthening tools: 12 common applications and a visual framework. Glob Health Sci Pract.

[6] World Health Organization (2021). Global strategy on digital health 2020-2025.

[7] Ochieng’ S, Hariharan N, Abuya T (2024). Exploring the implementation of an SMS-based digital health tool on maternal and infant health in informal settlements. BMC Pregnancy Childbirth.

[8] Onigbogi O, Ojo OY, Kinnunen U-M (2025). Mobile health interventions on vaccination coverage among children under 5 years of age in Low and Middle-Income countries; a scoping review. Front Public Health.

[9] Shartyanie NP, Hanifa IN, Khan N (2025). Digital Health Interventions in Emergency Obstetric and Newborn Care Services in Low- and Middle-Income Countries: Scoping Review. J Med Internet Res.

[10] Harrington EK, Drake AL, Matemo D (2019). An mHealth SMS intervention on Postpartum Contraceptive Use Among Women and Couples in Kenya: A Randomized Controlled Trial. Am J Public Health.

[11] Huang Y-Y, Wang R, Huang W-P (2024). Effects of a Smartphone-Based Breastfeeding Coparenting Intervention Program on Breastfeeding-Related Outcomes in Couples During First Pregnancy: Randomized Controlled Trial. J Med Internet Res.

[12] Kante M, Målqvist M (2025). Effectiveness of SMS-based interventions in enhancing antenatal care in developing countries: a systematic review. BMJ Open.

[13] Leeuw K A systematic review of digital health tools used for decision support by frontline health workers (FLHWs) in low- and middle- income countries (LMICs).

[14] Braun R, Catalani C, Wimbush J (2013). Community health workers and mobile technology: a systematic review of the literature. PLoS One.

[15] Deodhar J (2002). Telemedicine by email--experience in neonatal care at a primary care facility in rural India. J Telemed Telecare.

[16] Mohamed H, Ismail A, Sutan R (2025). A scoping review of digital technologies in antenatal care: recent progress and applications of digital technologies. BMC Pregnancy Childbirth.

[17] Sato R, Metiboba L, Galadanchi JA (2023). Cost analysis of an innovative eHealth program in Nigeria: a case study of the vaccine direct delivery system. BMC Public Health.

[18] Knop MR, Nagashima-Hayashi M, Lin R (2024). Impact of mHealth interventions on maternal, newborn, and child health from conception to 24 months postpartum in low- and middle-income countries: a systematic review. BMC Med.

[19] Peri SS, Bagchi AD, Baveja A (2022). A Systematic Review of the Effectiveness of Telemedicine in Reproductive and Neonatal Health in Rural and Low-Income Areas in India. *Telemed J E Health*.

[20] Nathan JJ, Abdullah A, Evans J (2025). Scaling digital health in low- and middle-income countries: lessons from Malaysia’s cross-sector capacity-building approach. J Glob Health.

[21] Mosadeghrad AM, Jaafaripooyan E, Zamandi M (2022). Economic Evaluation of Health Interventions: A Critical Review. Iran J Public Health.

[22] Shamseer L, Moher D, Clarke M (2015). Preferred reporting items for systematic review and meta-analysis protocols (PRISMA-P) 2015: elaboration and explanation. BMJ.

[23] Husereau D, Drummond M, Augustovski F (2022). Consolidated Health Economic Evaluation Reporting Standards 2022 (CHEERS 2022) Statement: Updated Reporting Guidance for Health Economic Evaluations. Value Health.

[24] Drummond ME, Sculpher MJ, Torrance GW (2005). Methods for the economic evaluation of health care programmes.

[25] Doran CM (2008). Economic evaluation of interventions to treat opiate dependence : a review of the evidence. Pharmacoeconomics.

[26] Nixon J, Khan KS, Kleijnen J (2001). Summarising economic evaluations in systematic reviews: a new approach. BMJ.

