# Supplementary material for: Economic evaluations of digital health interventions on maternal, newborn and child health in low-income and middle-income countries: a systematic review protocol
Source: BMJ Open. 2026 May 7;16(5):e115990. doi: 10.1136/bmjopen-2025-115990 (PMC13157750; doi:10.1136/bmjopen-2025-115990)
Supplement: online supplemental file 1 [file bmjopen-16-5-s001.pdf]

PRISMA-P (Preferred Reporting Items for Systematic review and Meta-Analysis Protocols) 2015 checklist: recommended items to address in a systematic review protocol*

| Section and topic | Item No | Checklist item |
|---|---|---|
| ADMINISTRATIVE INFORMATION | | |
| Title: | | |
| Identification | 1a | This is a protocol for a systematic review entitled "Systematic Review of Economic Evaluations of Digital Health Interventions on Maternal, Newborn and Child Health in Low- and Middle-income Countries" |
| Update | 1b | Not applicable. |
| Registration | 2 | PROSPERO ID: CRD420251125682 |
| Authors: | | |
| Contact | 3a | All names, institutional affiliation, e-mail address of all protocol authors have been provide and the physical mailing address of corresponding author has been provided on title page (Line 3-9). |
| Contributions | 3b | In "Contributors" section (Line 310-314). |
| Amendments | 4 | Any amendments will be reported in the final manuscript and updated in PROSPERO. |
| Support: | | |
| Sources | 5a | See line 315-317. |
| Sponsor | 5b | See line 315-317. |
| Role of sponsor or funder | 5c | The funder had no role in the design of this review, the development of the protocol, data collection, analysis, interpretation of findings, or the decision to submit this work for publication. |
| INTRODUCTION | | |
| Rationale | 6 | "Introduction" section describes burden of MNCH, digital health potential, gaps in economic evidence (Line 67-110). |
| Objectives | 7 | "Introduction" section describes the specific objectives of this review (Line 111-122). |
| METHODS | | |
| Eligibility criteria | 8 | Detailed under Inclusion & Exclusion Criteria: Population, Intervention, Comparator, Outcome, Study design, Context (Line 169-199). |

| | | |
|---|---|---|
| Information sources | 9 | In "Data source and search strategy" section (Line 128-156). |
| Search strategy | 10 | Full search strings included in Supplementary B. |
| Study records: | | |
| Data management | 11a | Covidence + Excel extraction sheet |
| Selection process | 11b | "Study selection" section: three independent reviewers, two stages (Line 200-209). |
| Data collection process | 11c | "Data extraction" section: pilot test, three independent reviewers, consensus process (Line 210-223). |
| Data items | 12 | Citation, country, income level, design, analytic approach, time horizon, currency/year, discount rate, intervention, comparator, cost outcomes, CE outcomes, etc (Line 210-223). |
| Outcomes and prioritization | 13 | ICERs, cost per DALY, cost per life saved, NMB, cost outcomes, cost drivers. |
| Risk of bias in individual studies | 14 | Drummond Checklist (Line 224-236). |
| Data synthesis | 15a | Meta-analysis not feasible; narrative synthesis planned (Line 237-269). |
| | 15b | Not applicable. |
| | 15c | Not applicable. |
| | 15d | Detailed in "Strategy for data synthesis". |
| Meta-bias(es) | 16 | Not applicable. |
| Confidence in cumulative evidence | 17 | *No formal overall certainty grading will be applied*. The review will comment narratively on methodological quality and reporting quality. |

* It is strongly recommended that this checklist be read in conjunction with the PRISMA-P Explanation and Elaboration (cite when available) for important clarification on the items. Amendments to a review protocol should be tracked and dated. The copyright for PRISMA-P (including checklist) is held by the PRISMA-P Group and is distributed under a Creative Commons Attribution Licence 4.0.

*From: Shamseer L, Moher D, Clarke M, Ghersi D, Liberati A, Petticrew M, Shekelle P, Stewart L, PRISMA-P Group. Preferred reporting items for systematic review and meta-analysis protocols (PRISMA-P) 2015: elaboration and explanation. BMJ. 2015 Jan 2;349(jan02 1):g7647.*
