# Supplementary material for: Economic evaluations of digital health interventions on maternal, newborn and child health in low-income and middle-income countries: a systematic review protocol
Source: BMJ Open. 2026 May 7;16(5):e115990. doi: 10.1136/bmjopen-2025-115990 (PMC13157750; doi:10.1136/bmjopen-2025-115990)
Supplement: online supplemental file 2 [file bmjopen-16-5-s002.pdf]

Search Strategy for PubMed：

	Topics	Search Term
1	Mobile Health	("Telemedicine"[Mesh] OR "Mobile Applications"[Mesh] OR "Smartphone"[Mesh] OR "Cell Phone"[Mesh] OR "Computers, Handheld"[Mesh] OR "Internet"[Mesh] OR "Text Messaging"[Mesh] OR "Remote Sensing Technology"[Mesh] OR "Wearable Electronic Devices"[Mesh] OR telemedicine[tiab] OR telehealth[tiab] OR "tele health"[tiab] OR telecare[tiab] OR "tele care"[tiab] OR "digital health"[tiab] OR "mobile health"[tiab] OR mhealth[tiab] OR "m-health"[tiab] OR ehealth[tiab] OR "e-health"[tiab] OR "electronic health"[tiab] OR "mobile app*"[tiab] OR "health app*"[tiab] OR "medical app*"[tiab] OR "smart phone*"[tiab] OR smartphone*[tiab] OR "cell phone*"[tiab] OR cellphone*[tiab] OR "tablet computer*"[tiab] OR tablet*[tiab] OR ipad*[tiab] OR "text messag*"[tiab] OR sms[tiab] OR "short messag*"[tiab] OR mms[tiab] OR "wearable*"[tiab] OR "fitness tracker*"[tiab] OR "activity monitor*"[tiab] OR "remote monitor*"[tiab] OR "remote consult*"[tiab] OR "virtual consult*"[tiab] OR "chatbot*"[tiab] OR "voice assist*"[tiab] OR "interactive voice"[tiab] OR ivr[tiab])
2	Maternal, Neonatal and Child Health Care	
	2.1 Maternal Health & Complications	("Maternal Health"[Mesh] OR "Midwifery"[Mesh] OR "Pregnancy"[Mesh] OR "Delivery, Obstetric"[Mesh] OR "Postpartum Period"[Mesh] OR "Family Planning Services"[Mesh] OR "Contraception"[Mesh] OR "Pregnancy Complications"[Mesh] OR "Maternal Mortality"[Mesh] OR "Abortion, Induced"[Mesh] OR "Abortion, Spontaneous"[Mesh] OR "Diabetes, Gestational"[Mesh] OR "Hypertension, Pregnancy-Induced"[Mesh] OR "Obesity, Maternal"[Mesh] OR "Depression, Postpartum"[Mesh] OR matern*[tiab] OR pregnan*[tiab] OR gestation*[tiab] OR postpartum[tiab] OR postnatal[tiab] OR antenatal[tiab] OR prenatal[tiab] OR perinatal[tiab] OR "intrapartum"[tiab] OR midwife*[tiab] OR "birth attendant*"[tiab] OR labor[tiab] OR labour[tiab] OR childbirth*[tiab] OR "obstetric*"[tiab] OR "family planning"[tiab] OR contracept*[tiab] OR abortion*[tiab] OR "gestational diabetes"[tiab] OR "pregnancy induced hypertension"[tiab] OR eclampsia[tiab] OR "pre-eclampsia"[tiab] OR "maternal obesity"[tiab] OR "maternal depression"[tiab] OR "postpartum depression"[tiab] OR "antenatal depression"[tiab])
	2.2 Infant & Health & Child Health & Complications	("Infant Health"[Mesh] OR "Child Health"[Mesh] OR "Infant, Newborn"[Mesh] OR "Infant Mortality"[Mesh] OR "Child Mortality"[Mesh] OR "Birth Weight"[Mesh] OR "Infant, Low Birth Weight"[Mesh] OR "Breast Feeding"[Mesh] OR "Fetal Growth Retardation"[Mesh] OR infant*[tiab] OR baby[tiab] OR babies[tiab] OR newborn*[tiab] OR neonat*[tiab] OR paediatr*[tiab] OR pediatr*[tiab] OR child*[tiab] OR toddler*[tiab] OR "breastfeeding"[tiab] OR "child survival"[tiab] OR "infant survival"[tiab] OR "child death*"[tiab] OR "infant death*"[tiab] OR "child mortality"[tiab] OR "infant mortality"[tiab] OR stillbirth*[tiab] OR "birth weight"[tiab] OR "birthweight"[tiab] OR "fetal growth retardation"[tiab] OR "foetal growth retardation"[tiab] OR "intrauterine growth retardation"[tiab] OR "fetal growth restriction"[tiab])

	2.3 Infectious Disease Transmission & Vaccination & major MNCH-related Diseases	("Infectious Disease Transmission, Vertical"[Mesh] OR "Immunization"[Mesh] OR "Vaccination"[Mesh] OR "Sexually Transmitted Diseases"[Mesh] OR "vertical transmission"[tiab] OR "mother to child transmission"[tiab] OR "mother-to-child transmission"[tiab] OR "maternal immunization"[tiab] OR "maternal immunisation"[tiab] OR "maternal vaccination"[tiab] OR "infant vaccination"[tiab] OR "infant immunization"[tiab] OR "infant immunisation"[tiab] OR "childhood immunization"[tiab] OR "childhood immunisation"[tiab] OR "childhood vaccination"[tiab] OR "sexually transmitted disease*"[tiab] OR "sexually transmitted infection*"[tiab] OR STD[tiab] OR STI[tiab] OR HIV[tiab] OR "human immunodeficiency virus"[tiab] OR syphilis[tiab] OR malaria[tiab] OR sepsis[tiab] OR diarrhea[tiab] OR diarrhoea[tiab] OR pneumonia[tiab] OR measles[tiab] OR tuberculosis[tiab])
	2.4 Cross-cutting & Health Systerm Terms	("Maternal-Child Health Services"[Mesh] OR "Maternal Health Services"[Mesh] OR "Child Health Services"[Mesh] OR "Prenatal Education"[Mesh] OR "Maternal Child Health"[tiab] OR "Maternal and Child Health"[tiab] OR "mother and child"[tiab] OR "MNCH"[tiab] OR " Maternal Newborn and Child Health"[tiab] OR "MCH"[tiab] OR "continuum of care"[tiab] OR "prenatal education"[tiab] OR "antenatal education"[tiab])
	2.5 Evidence-based MNCH Interventions	("kangaroo mother care"[tiab] OR "kangaroo care"[tiab] OR KMC[tiab] OR "oral rehydration therapy"[tiab]<br>OR "oral rehydration solution"[tiab] OR ORT[tiab] OR ORS[tiab] OR "exclusive breastfeeding"[tiab]<br>OR "breast feeding"[MeSH] OR "antenatal care"[MeSH]<br>OR "postnatal care"[MeSH] OR "skilled birth attendance"[tiab] OR "facility delivery"[tiab])
3	Economic Evaluation	("economic evaluation"[tiab] OR "economic analysis"[tiab] OR "cost effectiveness"[tiab] OR "cost utility"[tiab] OR "cost benefit"[tiab] OR "cost analysis"[tiab] OR "cost minimization"[tiab] OR "cost consequence"[tiab] OR "budget impact"[tiab] OR "health economic*"[tiab] OR "value for money"[tiab] OR cost*[tiab] OR economic*[tiab] OR price*[tiab] OR pricing*[tiab]) OR ("Cost-Benefit Analysis"[MeSH] OR "Costs and Cost Analysis"[MeSH] OR "Health Care Costs"[MeSH] OR "Economics, Medical"[MeSH] OR "Economics, Pharmaceutical"[MeSH])

Adapted search strategy for Embase:

	Topics	Search Term
1	Mobile Health	('telemedicine'/exp OR 'mobile application'/exp OR 'smartphone'/exp OR 'mobile phone'/exp OR 'personal digital assistant'/exp OR 'Internet'/exp OR 'text messaging'/exp OR 'remote sensing'/exp OR 'wearable electronic device'/ OR telemedicine:ti,ab OR telehealth:ti,ab OR "tele health":ti,ab OR telecare:ti,ab OR "tele care":ti,ab OR "digital health":ti,ab OR "mobile health":ti,ab OR mhealth:ti,ab OR m-health:ti,ab OR ehealth:ti,ab OR e-health:ti,ab OR "electronic health":ti,ab OR ("mobile" NEXT app*):ti,ab OR ("health" NEXT app*):ti,ab OR ("medical" NEXT app*):ti,ab OR ("smart" NEXT phone*):ti,ab OR smartphone*:ti,ab OR ("cell" NEXT phone*):ti,ab OR cellphone*:ti,ab OR ("tablet" NEXT computer*):ti,ab OR tablet*:ti,ab OR ipad*:ti,ab OR ("text" NEXT messag*):ti,ab OR sms:ti,ab OR ("short" NEXT messag*):ti,ab OR mms:ti,ab OR wearable*:ti,ab OR ("fitness" NEXT tracker*):ti,ab OR ("activity" NEXT monitor*):ti,ab OR ("remote" NEXT monitor*):ti,ab OR ("remote" NEXT consult*):ti,ab OR ("virtual" NEXT consult*):ti,ab OR chatbot*:ti,ab OR ("voice" NEXT assist*):ti,ab OR "interactive voice":ti,ab OR ivr:ti,ab)
2	Maternal, Neonatal I and Child Health Care	
	2.1 Maternal Health & Complications	('maternal welfare'/exp OR 'midwife'/exp OR 'pregnancy'/exp OR 'obstetric delivery'/exp OR 'puerperium'/exp OR 'family planning'/exp OR 'contraception'/exp OR 'pregnancy complication'/exp OR 'maternal mortality'/exp OR 'induced abortion'/exp OR 'spontaneous abortion'/exp OR 'gestational diabetes'/exp OR 'maternal hypertension'/exp OR 'maternal obesity'/exp OR 'postnatal depression'/exp OR matern*:ti,ab OR pregnan*:ti,ab OR gestation*:ti,ab OR postpartum:ti,ab OR postnatal:ti,ab OR antenatal:ti,ab OR prenatal:ti,ab OR perinatal:ti,ab OR intrapartum:ti,ab OR midwife*:ti,ab OR 'birth attendant*':ti,ab OR labor:ti,ab OR labour:ti,ab OR childbirth*:ti,ab OR obstetric*:ti,ab OR 'family planning':ti,ab OR contracept*:ti,ab OR abortion*:ti,ab OR 'gestational diabetes':ti,ab OR 'pregnancy induced hypertension':ti,ab OR eclampsia:ti,ab OR pre-eclampsia:ti,ab OR 'maternal obesity':ti,ab OR 'maternal depression':ti,ab OR 'postpartum depression':ti,ab OR 'antenatal depression':ti,ab)
	2.2 Infant & Health & Child Health & Complications	('child health'/exp OR 'child health'/exp OR 'newborn'/exp OR 'infant mortality'/exp OR 'childhood mortality'/exp OR 'birth weight'/exp OR 'low birth weight'/exp OR 'breast feeding'/exp OR 'intrauterine growth retardation'/exp OR infant*:ti,ab OR baby:ti,ab OR babies:ti,ab OR newborn*:ti,ab OR neonat*:ti,ab OR paediatr*:ti,ab OR pediatr*:ti,ab OR child*:ti,ab OR toddler*:ti,ab OR breastfeeding:ti,ab OR 'child survival':ti,ab OR 'infant survival':ti,ab OR 'child death*':ti,ab OR 'infant death*':ti,ab OR 'child mortality':ti,ab OR 'infant mortality':ti,ab OR stillbirth*:ti,ab OR 'birth weight':ti,ab OR birthweight:ti,ab OR 'fetal growth retardation':ti,ab OR 'foetal growth retardation':ti,ab OR 'intrauterine growth retardation':ti,ab OR 'fetal growth restriction':ti,ab)
	2.3 Infectious Disease Transmission &	('vertical transmission'/exp OR 'immunization'/exp OR 'vaccination'/exp OR 'sexually transmitted disease'/exp OR 'vertical transmission':ti,ab OR 'mother to child transmission':ti,ab OR 'mother-to-child transmission':ti,ab

	Vaccination & major MNCH-related Diseases	OR 'maternal immunization':ti,ab OR 'maternal immunisation':ti,ab OR 'maternal vaccination':ti,ab OR 'infant vaccination':ti,ab OR 'infant immunization':ti,ab OR 'infant immunisation':ti,ab OR 'childhood immunization':ti,ab OR 'childhood immunisation':ti,ab OR 'childhood vaccination':ti,ab OR 'sexually transmitted disease*':ti,ab OR 'sexually transmitted infection*':ti,ab OR STD:ti,ab OR STI:ti,ab OR HIV:ti,ab OR 'human immunodeficiency virus':ti,ab OR syphilis:ti,ab OR malaria:ti,ab OR sepsis:ti,ab OR diarrhea:ti,ab OR diarrhoea:ti,ab OR pneumonia:ti,ab OR measles:ti,ab OR tuberculosis:ti,ab)
	2.4 Cross-cutting & Health Systerm Terms	('maternal child health care'/exp OR 'maternal health service'/exp OR 'child health care'/exp OR 'childbirth education'/exp OR 'Maternal Child Health':ti,ab OR 'Maternal and Child Health':ti,ab OR 'mother and child':ti,ab OR MNCH:ti,ab OR 'Maternal Newborn and Child Health':ti,ab OR MCH:ti,ab OR 'continuum of care':ti,ab OR 'prenatal education':ti,ab OR 'antenatal education':ti,ab)
	2.5 Evidence-based MNCH Interventions	('breast feeding'/exp OR 'postnatal care'/exp OR 'kangaroo mother care':ti,ab OR 'kangaroo care':ti,ab OR KMC:ti,ab OR 'oral rehydration therapy':ti,ab OR 'oral rehydration solution':ti,ab OR ORT:ti,ab OR ORS:ti,ab OR 'exclusive breastfeeding':ti,ab OR 'skilled birth attendance':ti,ab OR 'facility delivery':ti,ab)
3	Economic Evaluation	("economic evaluation":ti,ab OR "economic analysis":ti,ab OR "cost effectiveness":ti,ab OR cost-effective:ti,ab OR cost-effectiveness:ti,ab OR "cost effectiveness":ti,ab OR "cost utility":ti,ab OR "cost benefit":ti,ab OR "cost analysis":ti,ab OR "cost minimization":ti,ab OR "cost consequence":ti,ab OR "budget impact":ti,ab OR ("health" NEXT economic*):ti,ab OR "value for money":ti,ab OR 'cost benefit analysis'/exp OR 'health care cost'/exp OR 'health economics'/exp OR 'pharmacoeconomics'/exp)

Adapted search strategy for Scopus:

	Topics	Search Term
1	Mobile Health	((TITLE-ABS (telemedicine) OR TITLE-ABS (telehealth) OR TITLE-ABS ("tele health") OR TITLE-ABS (telecare) OR TITLE-ABS ("tele care") OR TITLE-ABS ("digital health") OR TITLE-ABS ("mobile health") OR TITLE-ABS (mhealth) OR TITLE-ABS (m-health) OR TITLE-ABS (ehealth) OR TITLE-ABS (e-health) OR TITLE-ABS ("electronic health") OR TITLE-ABS ("mobile app*") OR TITLE-ABS ("health app*") OR TITLE-ABS ("medical app*") OR TITLE-ABS ("smart phone*") OR TITLE-ABS (smartphone*) OR TITLE-ABS ("cell phone*") OR TITLE-ABS (cellphone*) OR TITLE-ABS ("tablet computer*") OR TITLE-ABS (tablet*) OR TITLE-ABS (ipad*) OR TITLE-ABS ("text messag*") OR TITLE-ABS (sms) OR TITLE-ABS ("short messag*") OR TITLE-ABS (mms) OR TITLE-ABS (wearable*) OR TITLE-ABS ("fitness tracker*") OR TITLE-ABS ("activity monitor*") OR TITLE-ABS ("remote monitor*") OR TITLE-ABS ("remote consult*") OR TITLE-ABS ("virtual consult*") OR TITLE-ABS (chatbot*) OR TITLE-ABS ("voice assist*") OR TITLE-ABS ("interactive voice") OR TITLE-ABS (ivr)
2	Maternal, Neonatal and Child Health Care	
	2.1 Maternal Health & Complications	(TITLE-ABS (matern*) OR TITLE-ABS (pregnan*) OR TITLE-ABS (gestation*) OR TITLE-ABS (postpartum) OR TITLE-ABS (postnatal) OR TITLE-ABS (antenatal) OR TITLE-ABS (prenatal) OR TITLE-ABS (perinatal) OR TITLE-ABS (intrapartum) OR TITLE-ABS (midwife*) OR TITLE-ABS ("birth attendant*") OR TITLE-ABS (labor) OR TITLE-ABS (labour) OR TITLE-ABS (childbirth*) OR TITLE-ABS (obstetric*) OR TITLE-ABS ("family planning") OR TITLE-ABS (contracept*) OR TITLE-ABS (abortion*) OR TITLE-ABS ("gestational diabetes") OR TITLE-ABS ("pregnancy induced hypertension") OR TITLE-ABS (eclampsia) OR TITLE-ABS (pre-eclampsia) OR TITLE-ABS ("maternal obesity") OR TITLE-ABS ("maternal depression") OR TITLE-ABS ("postpartum depression") OR TITLE-ABS ("antenatal depression")
	2.2 Infant & Health & Child Health & Complications	(TITLE-ABS (infant*) OR TITLE-ABS (baby) OR TITLE-ABS (babies) OR TITLE-ABS (newborn*) OR TITLE-ABS (neonat*) OR TITLE-ABS (paediatr*) OR TITLE-ABS (pediatr*) OR TITLE-ABS (child*) OR TITLE-ABS (toddler*) OR TITLE-ABS (breastfeeding) OR TITLE-ABS ("child survival") OR TITLE-ABS ("infant survival") OR TITLE-ABS ("child death*") OR TITLE-ABS ("infant death*") OR TITLE-ABS ("child mortality") OR TITLE-ABS ("infant mortality") OR TITLE-ABS (stillbirth*) OR TITLE-ABS ("birth weight") OR TITLE-ABS (birthweight) OR TITLE-ABS ("fetal growth retardation") OR TITLE-ABS ("foetal growth retardation") OR TITLE-ABS ("intrauterine growth retardation") OR TITLE-ABS ("fetal growth restriction")

	2.3 Infectious Disease Transmission & Vaccination & major MNCH-related Diseases	(TITLE-ABS ("vertical transmission") OR TITLE-ABS ("mother to child transmission") OR TITLE-ABS ("mother-to-child transmission") OR TITLE-ABS ("maternal immunization") OR TITLE-ABS ("maternal immunisation") OR TITLE-ABS ("maternal vaccination") OR TITLE-ABS ("infant vaccination") OR TITLE-ABS ("infant immunization") OR TITLE-ABS ("infant immunisation") OR TITLE-ABS ("childhood immunization") OR TITLE-ABS ("childhood immunisation") OR TITLE-ABS ("childhood vaccination") OR TITLE-ABS ("sexually transmitted disease*") OR TITLE-ABS ("sexually transmitted infection*") OR TITLE-ABS (STD) OR TITLE-ABS (STI) OR TITLE-ABS (HIV) OR TITLE-ABS ("human immunodeficiency virus") OR TITLE-ABS (syphilis) OR TITLE-ABS (malaria) OR TITLE-ABS (sepsis) OR TITLE-ABS (diarrhea) OR TITLE-ABS (diarrhoea) OR TITLE-ABS (pneumonia) OR TITLE-ABS (measles) OR TITLE-ABS (tuberculosis)
	2.4 Cross-cutting & Health Systerm Terms	(TITLE-ABS ("Maternal Child Health") OR TITLE-ABS ("Maternal and Child Health") OR TITLE-ABS ("mother and child") OR TITLE-ABS (MNCH) OR TITLE-ABS ("Maternal Newborn and Child Health") OR TITLE-ABS (MCH) OR TITLE-ABS ("continuum of care") OR TITLE-ABS ("prenatal education") OR TITLE-ABS ("antenatal education")
	2.5 Evidence-based MNCH Interventions	(TITLE-ABS ("kangaroo mother care") OR TITLE-ABS ("kangaroo care") OR TITLE-ABS (KMC) OR TITLE-ABS ("oral rehydration therapy") OR TITLE-ABS ("oral rehydration solution") OR TITLE-ABS (ORT) OR TITLE-ABS (ORS) OR TITLE-ABS ("exclusive breastfeeding") OR TITLE-ABS ("skilled birth attendance") OR TITLE-ABS ("facility delivery")
3	Economic Evaluation	(TITLE-ABS ("economic evaluation") OR TITLE-ABS ("economic analysis") OR TITLE-ABS ("cost effectiveness") OR TITLE-ABS (cost-effective) OR TITLE-ABS (cost-effectiveness) OR TITLE-ABS ("cost utility") OR TITLE-ABS ("cost benefit") OR TITLE-ABS ("cost analysis") OR TITLE-ABS ("cost minimization") OR TITLE-ABS ("cost consequence") OR TITLE-ABS ("budget impact") OR TITLE-ABS ("health economic*") OR TITLE-ABS ("value for money")

Adapted search strategy for Web of Science:

	Topics	Search Term
1	Mobile Health	(TS=telemedicine OR TS=telehealth OR TS="tele health" OR TS=telecare OR TS="tele care" OR TS="digital health" OR TS="mobile health" OR TS=mhealth OR TS="m-health" OR TS=ehealth OR TS="e-health" OR TS="electronic health" OR TS="mobile app*" OR TS="health app*" OR TS="medical app*" OR TS="smart phone*" OR TS=smartphone* OR TS="cell phone*" OR TS=cellphone* OR TS="tablet computer*" OR TS=tablet* OR TS=ipad* OR TS="text messag*" OR TS=sms OR TS="short messag*" OR TS=mms OR TS=wearable* OR TS="fitness tracker*" OR TS="activity monitor*" OR TS="remote monitor*" OR TS="remote consult*" OR TS="virtual consult*" OR TS=chatbot* OR TS="voice assist*" OR TS="interactive voice" OR TS=ivr)
2	Maternal, Neonatal I	and Child Health Care
	2.1 Maternal Health & Complications	(TS=matern* OR TS=pregnan* OR TS=gestation* OR TS=postpartum OR TS=postnatal OR TS=antenatal OR TS=prenatal OR TS=perinatal OR TS="intrapartum" OR TS=midwife* OR TS="birth attendant*" OR TS=labor OR TS=labour OR TS=childbirth* OR TS="obstetric*" OR TS="family planning" OR TS=contracept* OR TS=abortion* OR TS="gestational diabetes" OR TS="pregnancy induced hypertension" OR TS=eclampsia OR TS="pre-eclampsia" OR TS="maternal obesity" OR TS="maternal depression" OR TS="postpartum depression" OR TS="antenatal depression")
	2.2 Infant & Health & Child Health & Complications	(TS=infant* OR TS=baby OR TS=babies OR TS=newborn* OR TS=neonat* OR TS=paediatr* OR TS=pediatr* OR TS=child* OR TS=toddler* OR TS="breastfeeding" OR TS="child survival" OR TS="infant survival" OR TS="child death*" OR TS="infant death*" OR TS="child mortality" OR TS=stillbirth* OR TS="birth weight" OR TS=birthweight* OR TS="fetal growth retardation" OR TS="foetal growth retardation" OR TS="intrauterine growth retardation" OR TS="fetal growth restriction")
	2.3 Infectious Disease Transmission & Vaccination & major MNCH-related Diseases	(TS="vertical transmission" OR TS="mother to child transmission" OR TS="mother-to-child transmission" OR TS="maternal immunization" OR TS="maternal immunisation" OR TS="maternal vaccination" OR TS="infant vaccination" OR TS="childhood immunization" OR TS="sexually transmitted disease*" OR TS="sexually transmitted infection*" OR TS=STD OR TS=STI)
	2.4 Cross-cutting & Health Systerm Terms	(TS="Maternal Child Health" OR TS="Maternal and Child Health" OR TS="mother and child" OR TS=MNCH OR TS=" Maternal Newborn and Child Health" OR TS=MCH OR TS="continuum of care" OR TS="prenatal education" OR TS="antenatal education")
	2.5 Evidence-based MNCH Interventions	(TS="kangaroo mother care" OR TS="kangaroo care" OR TS=KMC OR TS="oral rehydration therapy" OR TS="oral rehydration solution" OR TS=ORT OR TS=ORS OR TS="exclusive breastfeeding" OR TS="skilled birth attendance" OR TS="facility delivery")
3	Economic Evaluation	(TS="economic evaluation" OR TS="economic evaluation" OR TS="economic analysis" OR TS="cost effectiveness" OR TS=cost-effective OR TS=cost-effectiveness

		OR TS="cost utility" OR TS="cost benefit" OR TS="cost analysis" OR TS="cost minimization" OR TS="cost consequence" OR TS="budget impact" OR TS="health economic*" OR TS="value for money")

Adapted search strategy for Cochrane library:

	Topics	Search Term
1	Mobile Health	(MeSH descriptor: [Telemedicine] explode all trees OR MeSH descriptor: [Mobile Applications] explode all trees OR MeSH descriptor: [Smartphone] explode all trees OR MeSH descriptor: [Cell Phone] explode all trees OR MeSH descriptor: [Computers, Handheld] explode all trees OR MeSH descriptor: [Internet] explode all trees OR MeSH descriptor: [Text Messaging] explode all trees OR MeSH descriptor: [Remote Sensing Technology] explode all trees OR MeSH descriptor: [Wearable Electronic Devices] explode all trees OR telemedicine:ti,ab,kw OR telehealth:ti,ab,kw OR “tele health”:ti,ab,kw OR telecare:ti,ab,kw OR “tele care”:ti,ab,kw OR “digital health”:ti,ab,kw OR “mobile health”:ti,ab,kw OR mhealth:ti,ab,kw OR “m-health”:ti,ab,kw OR ehealth:ti,ab,kw OR “e-health”:ti,ab,kw OR “electronic health”:ti,ab,kw OR “mobile app*”:ti,ab,kw OR “health app*”:ti,ab,kw OR “medical app*”:ti,ab,kw OR “smart phone*”:ti,ab,kw OR smartphone*:ti,ab,kw OR “cell phone*”:ti,ab,kw OR cellphone*:ti,ab,kw OR “tablet computer*”:ti,ab,kw OR tablet*:ti,ab,kw OR ipad*:ti,ab,kw OR “text messag*”:ti,ab,kw OR sms:ti,ab,kw OR “short messag*”:ti,ab,kw OR mms:ti,ab,kw OR wearable*:ti,ab,kw OR “fitness tracker*”:ti,ab,kw OR “activity monitor*”:ti,ab,kw OR “remote monitor*”:ti,ab,kw OR “remote consult*”:ti,ab,kw OR “virtual consult*”:ti,ab,kw OR chatbot*:ti,ab,kw OR “voice assist*”:ti,ab,kw OR “interactive voice”:ti,ab,kw OR ivr:ti,ab,kw)
2	Maternal, Neonatal and Child Health Care	
	2.1 Maternal Health & Complications	(MeSH descriptor: [Maternal Health] explode all trees OR MeSH descriptor: [Midwifery] explode all trees OR MeSH descriptor: [Pregnancy] explode all trees OR MeSH descriptor: [Delivery, Obstetric] explode all trees OR MeSH descriptor: [Postpartum Period] explode all trees OR MeSH descriptor: [Family Planning Services] explode all trees OR MeSH descriptor: [Contraception] explode all trees OR MeSH descriptor: [Pregnancy Complications] explode all trees OR MeSH descriptor: [Maternal Mortality] explode all trees OR MeSH descriptor: [Abortion, Induced] explode all trees OR MeSH descriptor: [Abortion, Spontaneous] explode all trees OR MeSH descriptor: [Diabetes, Gestational] explode all trees OR MeSH descriptor: [Hypertension, Pregnancy-Induced] explode all trees OR MeSH descriptor: [Obesity, Maternal] explode all trees OR MeSH descriptor: [Depression, Postpartum] explode all trees OR matern*:ti,ab,kw OR pregnan*:ti,ab,kw OR gestation*:ti,ab,kw OR postpartum:ti,ab,kw OR postnatal:ti,ab,kw OR antenatal:ti,ab,kw OR prenatal:ti,ab,kw OR perinatal:ti,ab,kw OR intrapartum:ti,ab,kw OR midwife*:ti,ab,kw OR “birth attendant*”:ti,ab,kw OR labor:ti,ab,kw OR labour:ti,ab,kw OR childbirth*:ti,ab,kw OR obstetric*:ti,ab,kw OR “family planning”:ti,ab,kw OR contracept*:ti,ab,kw OR abortion*:ti,ab,kw OR “gestational diabetes”:ti,ab,kw OR “pregnancy induced hypertension”:ti,ab,kw OR eclampsia:ti,ab,kw OR “pre-eclampsia”:ti,ab,kw OR “maternal obesity”:ti,ab,kw OR “maternal depression”:ti,ab,kw OR “postpartum depression”:ti,ab,kw OR “antenatal depression”:ti,ab,kw)

	2.2 Infant & Health & Child Health & Complications	(MeSH descriptor: [Infant Health] explode all trees OR MeSH descriptor: [Child Health] explode all trees OR MeSH descriptor: [Infant, Newborn] explode all trees OR MeSH descriptor: [Infant Mortality] explode all trees OR MeSH descriptor: [Child Mortality] explode all trees OR MeSH descriptor: [Birth Weight] explode all trees OR MeSH descriptor: [Infant, Low Birth Weight] explode all trees OR MeSH descriptor: [Breast Feeding] explode all trees OR MeSH descriptor: [Fetal Growth Retardation] explode all trees OR infant*:ti,ab,kw OR baby:ti,ab,kw OR babies:ti,ab,kw OR newborn*:ti,ab,kw OR neonat*:ti,ab,kw OR paediatr*:ti,ab,kw OR pediatr*:ti,ab,kw OR child*:ti,ab,kw OR toddler*:ti,ab,kw OR breastfeeding:ti,ab,kw OR "child survival":ti,ab,kw OR "infant survival":ti,ab,kw OR "child death*":ti,ab,kw OR "infant death*":ti,ab,kw OR "child mortality":ti,ab,kw OR "infant mortality":ti,ab,kw OR stillbirth*:ti,ab,kw OR "birth weight":ti,ab,kw OR birthweight:ti,ab,kw OR "fetal growth retardation":ti,ab,kw OR "foetal growth retardation":ti,ab,kw OR "intrauterine growth retardation":ti,ab,kw OR "fetal growth restriction":ti,ab,kw)
	2.3 Infectious Disease Transmission & Vaccination & major MNCH-related Diseases	(MeSH descriptor: [Infectious Disease Transmission, Vertical] explode all trees OR MeSH descriptor: [Immunization] explode all trees OR MeSH descriptor: [Vaccination] explode all trees OR MeSH descriptor: [Sexually Transmitted Diseases] explode all trees OR "vertical transmission":ti,ab,kw OR "mother to child transmission":ti,ab,kw OR "mother-to-child transmission":ti,ab,kw OR "maternal immunization":ti,ab,kw OR "maternal immunisation":ti,ab,kw OR "maternal vaccination":ti,ab,kw OR "infant vaccination":ti,ab,kw OR "infant immunization":ti,ab,kw OR "infant immunisation":ti,ab,kw OR "childhood immunization":ti,ab,kw OR "childhood immunisation":ti,ab,kw OR "childhood vaccination":ti,ab,kw OR "sexually transmitted disease*":ti,ab,kw OR "sexually transmitted infection*":ti,ab,kw OR STD:ti,ab,kw OR STI:ti,ab,kw OR HIV:ti,ab,kw OR "human immunodeficiency virus":ti,ab,kw OR syphilis:ti,ab,kw OR malaria:ti,ab,kw OR sepsis:ti,ab,kw OR diarrhea:ti,ab,kw OR diarrhoea:ti,ab,kw OR pneumonia:ti,ab,kw OR measles:ti,ab,kw OR tuberculosis:ti,ab,kw)
	2.4 Cross-cutting & Health Systerm Terms	(MeSH descriptor: [Maternal-Child Health Services] explode all trees OR MeSH descriptor: [Maternal Health Services] explode all trees OR MeSH descriptor: [Child Health Services] explode all trees OR MeSH descriptor: [Prenatal Education] explode all trees OR "Maternal Child Health":ti,ab,kw OR "Maternal and Child Health":ti,ab,kw OR "mother and child":ti,ab,kw OR MNCH:ti,ab,kw OR "Maternal Newborn and Child Health":ti,ab,kw OR MCH:ti,ab,kw OR "continuum of care":ti,ab,kw OR "prenatal education":ti,ab,kw OR "antenatal education":ti,ab,kw)
	2.5 Evidence-based MNCH Interventions	(MeSH descriptor: [Breast Feeding] explode all trees OR MeSH descriptor: [Postnatal Care] explode all trees OR "kangaroo mother care":ti,ab,kw OR "kangaroo care":ti,ab,kw OR KMC:ti,ab,kw OR "oral rehydration therapy":ti,ab,kw OR "oral rehydration solution":ti,ab,kw OR ORT:ti,ab,kw OR ORS:ti,ab,kw OR "exclusive breastfeeding":ti,ab,kw OR "skilled birth attendance":ti,ab,kw OR "facility delivery":ti,ab,kw)

3	Economic Evaluation	("economic evaluation":ti,ab,kw OR "economic analysis":ti,ab,kw OR "cost effectiveness":ti,ab,kw OR "cost-effective":ti,ab,kw OR "cost-effectiveness":ti,ab,kw OR "cost utility":ti,ab,kw OR "cost benefit":ti,ab,kw OR "cost analysis":ti,ab,kw OR "cost minimization":ti,ab,kw OR "cost consequence":ti,ab,kw OR "budget impact":ti,ab,kw OR "health economic*":ti,ab,kw OR "value for money":ti,ab,kw) OR (MeSH descriptor: [Cost-Benefit Analysis] explode all trees OR MeSH descriptor: [Costs and Cost Analysis] explode all trees OR MeSH descriptor: [Health Care Costs] explode all trees OR MeSH descriptor: [Economics, Medical] explode all trees OR MeSH descriptor: [Economics, Pharmaceutical] explode all trees)

Adapted strategy for CINAHL：

	Topics	Search Term
1	Mobile Health	MM “Telemedicine” OR MM “c” OR MM “Smartphone” OR MM “Cellular Phones” OR MM “Computers, Hand-held” OR MM “Internet” OR MM “Text Messaging” OR MM “Remote Consultation” OR XB (telemedicine OR telehealth OR "tele health" OR telecare OR "tele care" OR "digital health" OR "mobile health" OR mhealth OR m-health OR ehealth OR e-health OR "electronic health" OR "mobile app*" OR "health app*" OR "medical app*" OR "smart phone*" OR smartphone* OR "cell phone*" OR cellphone* OR "tablet computer*" OR tablet* OR ipad* OR "text messag*" OR sms OR "short messag*" OR mms OR wearable* OR "fitness tracker*" OR "activity monitor*" OR "remote monitor*" OR "remote consult*" OR "virtual consult*" OR chatbot* OR "voice assist*" OR "interactive voice" OR ivr)
2	Maternal, Neonatal and Child Health Care	
	2.1 Maternal Health & Complications	MM “Maternal Health” OR MM “Midwifery” OR MM “Pregnancy” OR MM “Delivery, Obstetric” OR MM “Postpartum Period” OR MM “Family Planning” OR MM “Contraception” OR MM “Pregnancy Complications” OR MM “Maternal Mortality” OR MM “Abortion, Induced” OR MM “Abortion, Spontaneous” OR MM “Diabetes, Gestational” OR MM “Hypertension, Pregnancy-Induced” OR MM “Obesity, Maternal” OR MM “Depression, Postpartum” OR XB (matern* OR pregnan* OR gestation* OR postpartum OR postnatal OR antenatal OR prenatal OR perinatal OR intrapartum OR midwife* OR "birth attendant*" OR labor OR labour OR childbirth* OR obstetric* OR "family planning" OR contracept* OR abortion* OR "gestational diabetes" OR "pregnancy induced hypertension" OR eclampsia OR pre-eclampsia OR "maternal obesity" OR "maternal depression" OR "postpartum depression" OR "antenatal depression")
	2.2 Infant & Health & Child Health & Complications	MM “Infant Health” OR MM “Child Health” OR MM “Infant, Newborn” OR MM “Infant Mortality” OR MM “Child Mortality” OR MM “Birth Weight” OR MM “Infant, Low Birth Weight” OR MM “Breast Feeding” OR MM “Fetal Growth Retardation” ORXB (infant* OR baby OR babies OR newborn* OR neonat* OR paediatr* OR pediatr* OR child* OR toddler* OR breastfeeding OR "child survival" OR "infant survival" OR "child death*" OR "infant death*" OR "child mortality" OR stillbirth* OR "birth weight" OR birthweight* OR "fetal growth retardation" OR "foetal growth retardation" OR "intrauterine growth retardation" OR "fetal growth restriction")
	2.3 Infectious Disease Transmission & Vaccination & major MNCH-related Diseases	MM “Communicable Disease” OR MM “Immunization” OR MM “Vaccination” OR MM “Sexually Transmitted Diseases” OR XB ("vertical transmission" OR "mother to child transmission" OR "mother-to-child transmission" OR "maternal immunization" OR "maternal immunisation" OR "maternal vaccination" OR "infant vaccination" OR "childhood immunization" OR "sexually transmitted disease*" OR "sexually transmitted infection*" OR STD OR STI)

	2.4 Cross-cutting & Health Systerm Terms	MM “Maternal-Child Health” OR MM “Maternal Health Services” OR MM “Child Health Services” OR MM “Childbirth Education” OR XB ("Maternal Child Health" OR "Maternal and Child Health" OR "mother and child" OR MNCH OR " Maternal Newborn and Child Health" OR MCH OR "continuum of care" OR "prenatal education" OR "antenatal education")
	2.5 Evidence-based MNCH Interventions	MM “Breast Feeding” OR MM “Prenatal Care” OR MM “Postnatal Care” OR XB ("kangaroo mother care" OR "kangaroo care" OR KMC OR "oral rehydration therapy" OR "oral rehydration solution" OR ORT OR ORS OR "exclusive breastfeeding" OR "skilled birth attendance" OR "facility delivery")
3	Economic Evaluation	MM “Cost Benefit Analysis” OR MM “Costs and Cost Analysis” OR MM “Health Care Costs” OR MM “Cost Effectiveness Analysis” OR MM “Economics, Pharmaceutical” OR XB ("economic evaluation" OR "economic analysis" OR "cost effectiveness" OR "cost-effective" OR "cost-effectiveness" OR "cost utility" OR "cost benefit" OR "cost analysis" OR "cost minimization" OR "cost consequence" OR "budget impact" OR "health economic*" OR "value for money")

Adapted strategy for PsycInfo：

	Topics	Search Term
1	Mobile Health	(telemedicine or telehealth or "tele health" or telecare or "tele care" or "digital health" or "mobile health" or mhealth or m-health or ehealth or e-health or "electronic health" or "mobile app*" or "health app*" or "medical app*" or "smart phone*" or smartphone* or "cell phone*" or cellphone* or "tablet computer*" or tablet* or ipad* or "text messag*" or sms or "short messag*" or mms or wearable* or "fitness tracker*" or "activity monitor*" or "remote monitor*" or "remote consult*" or "virtual consult*" or chatbot* or "voice assist*" or "interactive voice" or ivr)).ab. OR (telemedicine or telehealth or "tele health" or telecare or "tele care" or "digital health" or "mobile health" or mhealth or m-health or ehealth or e-health or "electronic health" or "mobile app*" or "health app*" or "medical app*" or "smart phone*" or smartphone* or "cell phone*" or cellphone* or "tablet computer*" or tablet* or ipad* or "text messag*" or sms or "short messag*" or mms or wearable* or "fitness tracker*" or "activity monitor*" or "remote monitor*" or "remote consult*" or "virtual consult*" or chatbot* or "voice assist*" or "interactive voice" or ivr)).ti.
2	Maternal, Neonatal and Child Health Care	
	2.1 Maternal Health & Complications	(matern* or pregnan* or gestation* or postpartum or postnatal or antenatal or prenatal or perinatal or intrapartum or midwife* or "birth attendant*" or labor or labour or childbirth* or obstetric* or "family planning" or contracept* or abortion* or "gestational diabetes" or "pregnancy induced hypertension" or eclampsia or pre-eclampsia or "maternal obesity" or "maternal depression" or "postpartum depression" or "antenatal depression").ab. OR (matern* or pregnan* or gestation* or postpartum or postnatal or antenatal or prenatal or perinatal or intrapartum or midwife* or "birth attendant*" or labor or labour or childbirth* or obstetric* or "family planning" or contracept* or abortion* or "gestational diabetes" or "pregnancy induced hypertension" or eclampsia or pre-eclampsia or "maternal obesity" or "maternal depression" or "postpartum depression" or "antenatal depression").ti.
	2.2 Infant & Health & Child Health & Complications	(infant* or baby or babies or newborn* or neonat* or paediatr* or pediatr* or child* or toddler* or breastfeeding or "child survival" or "infant survival" or "child death*" or "infant death*" or "child mortality" or stillbirth* or "birth weight" or birthweight* or "fetal growth retardation" or "foetal growth retardation" or "intrauterine growth retardation" or "fetal growth restriction").ab. OR (infant* or baby or babies or newborn* or neonat* or paediatr* or pediatr* or child* or toddler* or breastfeeding or "child survival" or "infant survival" or "child death*" or "infant death*" or "child mortality" or stillbirth* or "birth weight" or birthweight* or "fetal growth retardation" or "foetal growth retardation" or "intrauterine growth retardation" or "fetal growth restriction").ti.
	2.3 Infectious Disease Transmission & Vaccination & major MNCH-related Diseases	("vertical transmission" or "mother to child transmission" or "mother-to-child transmission" or "maternal immunization" or "maternal immunisation" or "maternal vaccination" or "infant vaccination" or "childhood immunization" or "sexually transmitted disease*" or "sexually transmitted infection*" or STD or STI).ab. OR ("vertical transmission" or "mother to child transmission" or "mother-to-child transmission" or

		"maternal immunization" or "maternal immunisation" or "maternal vaccination" or "infant vaccination" or "childhood immunization" or "sexually transmitted disease*" or "sexually transmitted infection*" or STD or STI).ti.
	2.4 Cross-cutting & Health Systerm Terms	("Maternal Child Health" or "Maternal and Child Health" or "mother and child" or MNCH or "Maternal Newborn and Child Health" or MCH or "continuum of care" or "prenatal education" or "antenatal education").ab. OR ("Maternal Child Health" or "Maternal and Child Health" or "mother and child" or MNCH or " Maternal Newborn and Child Health" or MCH or "continuum of care" or "prenatal education" or "antenatal education").ti.
	2.5 Evidence-based MNCH Interventions	("kangaroo mother care" OR "kangaroo care" OR KMC OR "oral rehydration therapy" OR "oral rehydration solution" OR ORT OR ORS OR "exclusive breastfeeding" OR "skilled birth attendance" OR "facility delivery").ab. OR ("kangaroo mother care" OR "kangaroo care" OR KMC OR "oral rehydration therapy" OR "oral rehydration solution" OR ORT OR ORS OR "exclusive breastfeeding" OR "skilled birth attendance" OR "facility delivery").ti.
3	Economic Evaluation	("economic evaluation" OR "economic analysis" OR "cost effectiveness" OR "cost-effective" OR "cost-effectiveness" OR "cost utility" OR "cost benefit" OR "cost analysis" OR "cost minimization" OR "cost consequence" OR "budget impact" OR "health economic*" OR "value for money").ab. OR ("economic evaluation" OR "economic analysis" OR "cost effectiveness" OR "cost-effective" OR "cost-effectiveness" OR "cost utility" OR "cost benefit" OR "cost analysis" OR "cost minimization" OR "cost consequence" OR "budget impact" OR "health economic*" OR "value for money").ti.

Adapted strategy for TRIP Pro：

	Topics	Search Term
1	Mobile Health	((title:telemedicine OR title:telehealth OR title:"tele health" OR title:telecare OR title: "tele care" OR title:"digital health" OR title:"mobile health" OR title:mhealth OR title: "m-health" OR title:ehealth OR title: "e-health" OR title:"electronic health" OR title:"mobile app*" OR title:"health app*" OR title:smartphone* OR title:"cell phone*" OR title:"tablet computer*" OR title:"tablet*" OR title:"text messag*" OR title:sms OR title:"short messag*" OR title:mms OR title:wearable* OR title:"fitness tracker*" OR title:"activity monitor*" OR title:"remote monitor*" OR title:"remote consult*" OR title:"virtual consult*" OR title:chatbot* OR title:"voice assist*" OR title:"interactive voice" OR title:ivr))
2	Maternal, Neonatal I and Child Health Care	
	2.1 Maternal Health & Complications	(title:matern* OR title:pregnan* OR title:gestation* OR title:postpartum OR title:postnatal OR title:antenatal OR title:prenatal OR title:perinatal OR title:intrapartum OR title:midwife* OR title:"birth attendant*" OR title:labor OR title:labour OR title:childbirth* OR title:obstetric* OR title:"family planning" OR title:contracept* OR title:abortion* OR title:"gestational diabetes" OR title "pregnancy induced hypertension" OR title:eclampsia OR title:"pre-eclampsia" OR title:"maternal obesity"OR title:"maternal depression" OR title:"postporturm depression" OR title:"antenatal depression")
	2.2 Infant & Health & Child Health & Complications	(title:infant* OR title:baby OR title:babies OR title:newborn* OR title:neonat* OR title:paediatr* OR title:pediatr* OR title:child* OR title:toddler* OR title:breastfeeding OR title:"child survival" OR title:"infant survival" OR title:"child death*" OR title:"infant death*" OR title:"child mortality" OR title:"infant mortality" OR title:stillbirth* OR title:"birth weight" OR title:birthweight* OR title:"fetal growth retardation" OR title:"foetal growth retardation" OR title:"intrauterine growth retardation" OR title:"fetal growth restriction")
	2.3 Infectious Disease Transmission & Vaccination & major MNCH-related Diseases	(title:"vertical transmission" OR title:"mother to child transmission" OR title:"mother-to-child transmission" OR title:"maternal immunization" OR title:"maternal immunisation" OR title:"maternal vaccination" OR title:"infant vaccination" OR title:"infant immunization" OR title:"infant immunisation" OR title:"childhood immunization" OR title:"childhood immunisation" OR title:"sexually transmitted disease*" OR title:"sexually transmitted infection*" OR title:STD OR title:STI OR title:HIV OR title:"human immunodeficiency virus" OR title: syphilis OR title: malaria OR title: sepsis OR title: disrrhea OR title: diarrhoea OR title: pneumonia OR title: measles OR title: tuberculosis)
	2.4 Cross-cutting & Health Systerm Terms	(title:"Maternal Child Health" OR title:"Maternal and Child Health" OR title:"Mother and Child" OR title:" Maternal Newborn and Child Health" OR title:MNCH OR title:MCH OR title:"continuum of care" OR title:"prenatal education" OR title:"antenatal education")
	2.5 Evidence-based MNCH Interventions	(title:" kangaroo mother care" OR title:" kangaroo care" OR title: KMC OR title:"oral rehydration therapy" OR title:" oral rehydration solution" OR title:ORT OR title:ORS OR title:"exclusive breastfeeding" OR title:"skilled birth attendance" OR title:"skilled birth attendance" OR title:"facility delivery")

3	Economic Evaluation	((title:"economic evaluation" OR title:"economic analysis" OR title:"cost effectiveness" OR title:"cost-effective" OR title:"cost-effectiveness" OR title:"cost utility" OR title:"cost benefit" OR title:"cost analysis" OR title:"cost minimization" OR title:"cost consequence" OR title:"budget impact" OR title:"health economic*" OR title:"value for money"))
