# Supplementary material for: Economic evaluations of digital health interventions on maternal, newborn and child health in low-income and middle-income countries: a systematic review protocol
Source: BMJ Open. 2026 May 7;16(5):e115990. doi: 10.1136/bmjopen-2025-115990 (PMC13157750; doi:10.1136/bmjopen-2025-115990)
Supplement: online supplemental file 3 [file bmjopen-16-5-s003.pdf]

Prompt 1 (Concept exploration)

Generate examples of digital health interventions that have been used in maternal, newborn, and child health. Include commonly used terminology in the literature (e.g., telemedicine, mHealth, mobile applications, remote monitoring). Return the results as a list of intervention types and related keywords that may be useful when developing a database search strategy.

Prompt 2 (Keyword expansion)

Based on the following research question:

"What is the cost-effectiveness of digital health interventions in maternal, newborn, and child health?", suggest additional keywords, synonyms, related terms, and alternative spellings for the concepts:

1) digital health

2) economic evaluation

3) maternal, newborn, and child health

Return the results grouped by concept.

Prompt 3 (Search strategy refinement)

Review the following search terms used in a systematic review search strategy [insert the search strategy developed by the research team and refined using AI-generated suggestions]. Identify any potential missing synonyms, related terms, or alternative spellings that may improve the sensitivity of the search strategy.

Prompt 4 (Parallel search and coverage check)

Based on the following research topic: "economic evaluation of digital health interventions in maternal, newborn, and child health," identify potentially relevant peer-reviewed studies published from 2000 to 2025. Focus on studies that evaluate the cost-effectiveness, cost-utility, cost-benefit, cost-outcome, or economic impact of digital health interventions (e.g., telemedicine, mHealth, mobile applications, remote monitoring) related to maternal, newborn, and child health.

Return a list of potentially relevant articles, including the study title, authors, year of publication, and doi.
