# Supplementary material for: Economic evaluations of digital health interventions on maternal, newborn and child health in low-income and middle-income countries: a systematic review protocol
Source: BMJ Open. 2026 May 7;16(5):e115990. doi: 10.1136/bmjopen-2025-115990 (PMC13157750; doi:10.1136/bmjopen-2025-115990)
Supplement: Supplementary file 4 [file bmjopen-2025-115990.reviewer_comments.pdf]

PEER REVIEW HISTORY

BMJ Open publishes all reviews undertaken for accepted manuscripts. Reviewers are asked to complete a checklist review form and are provided with free text boxes to elaborate on their assessment. These free text comments are reproduced below.

ARTICLE DETAILS

Title (Provisional)

Economic evaluations of digital health interventions on maternal, newborn, and child health in low-and middle-income countries: a systematic review protocol

Authors

Wu, Fei; Soun, Brenda; Zhan, Binqi; Morton, Alec; Yi, Siyan

VERSION 1 - REVIEW

**Reviewer**	**1**
**Name**	**Barbosa , Mariane**
**Affiliation**	**Universidade Federal de Minas Gerais, Pediatric Dentistry**
**Date**	**09-Mar-2026**
**COI**	**None**

I would like to thank the editors for the opportunity to review the manuscript entitled "Economic evaluations of digital health interventions on reproductive, maternal, newborn, and child health in low- and middle-income countries: a systematic review protocol." The manuscript presents a research protocol for a systematic review on digital health interventions related to reproductive, maternal, newborn, and child health (RMNCH). This is a relevant and timely topic, given the increasing implementation of digital technologies in health systems and the need for evidence on cost-effectiveness to inform policy decisions and resource allocation.

Overall, the protocol is well structured. The authors clearly define key methodological aspects, including the search strategy across eight databases, the time frame for study selection (2000–2025), the inclusion and exclusion criteria, the use of methodological guidelines (PRISMA and PRISMA-P), and the data extraction process. The authors also indicate that the protocol has been registered in PROSPERO, which strengthens the transparency and methodological rigor of the study.

However, some methodological aspects could be further clarified in the manuscript, particularly those related to the search strategy, the use of artificial intelligence tools, eligibility criteria, and the approach to economic evaluation. In this regard, I would like to suggest the following points for consideration:

1 - The authors are encouraged to provide a more detailed description of the search strategy for each database, including any adaptations made to account for the specific characteristics of each database.

2- Regarding the use of the AI-based Deep Research tool (ChatGPT, OpenAI), it would be helpful to clarify whether this tool will be used solely to expand or complement the search strategy. I suggest that the authors provide more details about the methodological procedure, including the prompts used and the workflow of how the tool will be applied. Since generative AI tools are not structured bibliographic databases, it is important to clarify how the reproducibility of this process will be ensured.

3 - Did the authors consider including grey literature, such as institutional or governmental reports? In economic evaluations, particularly in low- and middle-income countries, such documents may contain valuable information on intervention costs and implementation.

4 - Additional clarification would be helpful regarding how cost data will be adjusted over time, particularly in relation to inflation adjustments and currency conversion. Furthermore, it would be useful to explain in more detail how cost-effectiveness results will be compared across the included studies, given the potential heterogeneity in methods, indicators, and contexts.

In general, I believe the manuscript presents a relevant and well-designed protocol. Addressing the points raised above could further strengthen the methodological transparency and reproducibility of the proposed systematic review.

VERSION 1 - AUTHOR RESPONSE

Comment 1 - The authors are encouraged to provide a more detailed description of the search strategy for each database, including any adaptations made to account for the specific characteristics of each database.

Response

Thank you for this helpful suggestion. We have revised the Methods section to provide a more detailed description of the search strategy used for each database. Specifically, we have clarified how the search terms and syntax will be adapted to align with the indexing systems and features of each database. Medical Subject Headings (MeSH) will be used in PubMed, Emtree terms in Embase, and CINAHL Headings in CINAHL where appropriate. Database-specific field tags and operators (e.g., title/abstract fields and truncation symbols) will be applied accordingly. The complete search strategies for all databases have now been provided in the **supplementary materials B** to enhance transparency and reproducibility. These revisions have been incorporated into the manuscript (Lines 171–178).

Comment 2- Regarding the use of the AI-based Deep Research tool (ChatGPT, OpenAI), it would be helpful to clarify whether this tool will be used solely to expand or complement the search strategy. I suggest that the authors provide more details about

the methodological procedure, including the prompts used and the workflow of how the tool will be applied. Since generative AI tools are not structured bibliographic databases, it is important to clarify how the reproducibility of this process will be ensured.

Response

Thank you for this helpful suggestion. We have revised the Methods section to clarify the role of the AI-based tool in developing the search strategy. Specifically, the generative AI tool (ChatGPT, OpenAI) will be used only as a supplementary aid during the search strategy development phase to help identify additional keywords, synonyms, and related concepts relevant to the review topic. It will not be used as a primary method for identifying or retrieving studies and does not replace structured searches of bibliographic databases.

To ensure reproducibility, representative prompts and the AI-assisted workflow have been documented in the **supplementary materials C**. In addition, any potentially relevant studies suggested by the AI tool will be verified and retrieved through structured searches of the bibliographic databases. These revisions have been incorporated into the manuscript (Lines 179–217).

Comment 3 - Did the authors consider including grey literature, such as institutional or governmental reports? In economic evaluations, particularly in low- and middle-income countries, such documents may contain valuable information on intervention costs and implementation.

Response

Thank you for this important suggestion. We acknowledge that grey literature, such as institutional or governmental reports, may provide valuable information on intervention costs and implementation. However, this review will focus on peer-reviewed studies to ensure methodological transparency and consistency in the reporting of economic evaluation methods and outcomes. Peer-reviewed publications generally provide more detailed information on study design, costing methods, and analytical approaches, which is essential for assessing study quality and comparability across economic evaluations.

In addition, grey literature sources often vary substantially in reporting standards and methodological detail, which may limit the ability to systematically extract and compare economic outcomes across studies. Given these considerations and the scope of the current review, we decided to restrict the search to peer-reviewed literature. Nevertheless, we agree that grey literature may contain relevant insights, and this limitation has been acknowledged in the manuscript. Clarifications have been incorporated into the manuscript (Lines 227–229, 399-402).

Comment 4 - Additional clarification would be helpful regarding how cost data will be adjusted over time, particularly in relation to inflation adjustments and currency conversion. Furthermore, it would be useful to explain in more detail how cost-effectiveness results will be compared across the included studies, given the potential heterogeneity in methods, indicators, and contexts.

Response

We agree that additional clarification regarding the standardization of cost data across studies is important. We have revised the manuscript to provide further details on how cost estimates will be adjusted for inflation and converted to a common currency, improving comparability across studies conducted in different countries and time periods. Specifically, costs reported in different years will be adjusted to a common price year (2024) using appropriate country-specific inflation indices obtained from the World Bank. The year 2024 was selected as it is the most recent year available from the World Bank. Subsequently, cost values will be converted to United States dollars using exchange rates from International Financial Statistics.

We have also expanded the data synthesis section to provide a more detailed description of our approach. Cost-effectiveness outcomes, including incremental cost-effectiveness ratios (ICERs), will be synthesised narratively rather than quantitatively due to the high heterogeneity. Where feasible, ICERs will be compared across studies reporting similar outcome measures. In addition, we will use a permutation matrix adapted from the Joanna Briggs Institute guidelines to classify interventions by their relative costs and effects (e.g., higher, lower, or similar costs and outcomes compared with the comparator). This approach allows for a structured comparison of economic findings across heterogeneous studies. These revisions have been incorporated into the manuscript (Lines 330–345).

Summary of revisions

We thank the reviewer for the constructive and insightful comments. He have provided detailed search strategies for all databases, clarified the role and workflow of the AI-assisted approach with example prompts included in the supplementary materials, and justified the exclusion of grey literature while acknowledging this as a limitation. We have also strengthened the data synthesis section by providing detailed methods for cost standardization.

In addition, we have refined the scope of the review and updated the manuscript title to “Economic evaluations of digital health interventions on maternal, newborn, and child health in low- and middle-income countries: a systematic review protocol”, with the removal of “reproductive” to better align with the study objectives.
